# Opportunities to enhance assisted living facility care quality through interprofessional collaborative practice

**DOI:** 10.3389/fragi.2026.1870661

**Published:** 2026-10-02

**Authors:** David Hage, Rekha Pudur, Jan Golembiski

**Affiliations:** 1 Department of Social Work, Shady Rest Institute on Positive Aging, Marieb College of Health and Human Services, Florida Gulf Coast University, Fort Myers, FL, United States; 2 Department of Health Sciences, Shady Rest Institute on Positive Aging, Marieb College of Health and Human Services, Florida Gulf Coast University, Fort Myers, FL, United States; 3 Department of Social Work, Marieb College of Health and Human Services, Florida Gulf Coast University, Fort Myers, FL, United States

**Keywords:** assisted living, IPCP inter-professional collaborative practice, IPE, longterm care, quality improvement

## Abstract

Interprofessional Education (IPE) is increasingly recognized as a vital strategy for enhancing the quality of care in assisted living facility (ALF) settings. By fostering collaboration among healthcare professionals such as nurses, nurses’ aides, social workers, physicians, and administrators, IPE promotes a shared and more robust understanding of resident needs and care goals. This opinion article argues that greater integration of IPE and interprofessional collaborative practice (IPCP) into long-term care training programs can significantly improve communication, reduce errors, and enhance person-centered care. Assisted living communities face complex challenges, including managing chronic conditions, supporting cognitive issues, changes in resident acuity, and addressing psychosocial needs. IPCP equips staff with the skills to navigate these challenges collectively, leading to more coordinated and effective care. While barriers such as scheduling challenges and institutional silos frequently persist, the potential benefits of IPCP include broader integration of IPCP towards improved resident outcomes, resident and staff satisfaction, and operational efficiencies that warrant its broader adoption. This article calls for greater prioritization of IPCP in assisted living facility settings to enhance resident outcomes and staff collaboration.

## Introduction

ALFs are presently at a critical crossroads, shaped by a confluence of demographic transformations, escalating clinical complexities, and enduring workforce challenges, all of which are redefining its function and ability to deliver care (Harris-Kojetin et al., 2016). Increasing assisted facility utilization combined with clinical care challenges and resource constraints make care outcomes more complicated (Nilormi et al., 2024; Wobert et al., 2025). Addressing evolving demands necessitates a fundamentally collaborative approach rather than mere incremental changes alone. Interprofessional education (IPE), defined as learning that occurs when professionals from two or more disciplines learning with, from, and about one another, presents a collaborative practice pathway to enhance teamwork and improve care outcomes (WHO, 2010). In ALF settings, IPCP approaches to care could foster strategic educational and practice-focused collaboration among nurses, social workers, physical therapists, prescribers, administrators, dietary staff, and other care providers to meet residents’ multifaceted needs (Reeves et al., 2018). Although IPCP is commonly recognized for its value in more medically intensive settings, it remains underutilized in ALFs (Thistlethwaite, 2011).

## Demographic and clinical context

In the United States, the population aged 65 years and older is experiencing unprecedented growth. Projections indicate that by 2050, approximately 10,000 individuals will reach age 65 each day, representing roughly 20% of the total U.S. population and an additional 22% of the global population will exceed 60 years of age (Eggersdorfer et al., 2018; WHO, 2025). This accelerated aging trend not only increases the number of older adults, but also intensifies the complexity of their healthcare needs within residential long-term care environments (McSweeney-Feld et al., 2012). ALFs first emerged in the 1980’s to provide a person-centered approach to caring for the needs of older adults. Many people had an aversion to nursing homes but needed support with their ADL’s. Over time, ALF models became hospitality focused rather than healthcare focused. This made the ALF level of care more palatable for older adults. Unfortunately, this backfired as residents required a higher level of care that facilities were ill equipped to handle. Now ALFs frequently have resident populations with complex needs that often exceed the original intent of this level of care.

## The problem of increasing resident acuity

Traditionally, ALF’s have been designed as supportive, homelike environments that provide housing, regular staff supervision, and assistance with activities of daily living (ADLs), including bathing, dressing, grooming, and medication management (Crowley et al., 2022; Zimmerman et al., 2022). Unlike nursing homes, which are more medically oriented, ALFs have historically catered to individuals who require help with a range of daily tasks, but do not need continuous skilled nursing care (Crowley et al., 2022). However, this distinction is becoming increasingly nebulous. Currently, nearly 90% of ALF residents live with at least one chronic condition, such as cancer, dementia, diabetes, or heart disease among others (Boersma et al., 2020).

ALFs face a fundamental mismatch between the growing complexity of its residents’ care needs and the system’s capacity to support them (Zimmerman et al., 2022). Acuity creep refers to the phenomenon of creeping of care needs of residents in ALFs that often involve health, cognition, functional assistance needs, or other non-medical supervision needs that extend beyond the intended scope of ALF services and staffing capabilities (Moccia et al., 2021; Trainum et al., 2024; Trinkoff et al., 2020). Consequently, ALFs are now being called upon to deliver more complex and coordinated care than ever before (Mowbray et al., 2023).

ALFs serve a population with increasingly complex medical, cognitive and psychosocial needs (Zimmerman et al., 2022). Nationally, more than one-half of ALF residents are aged 85 or older, and chronic health conditions are highly typical. Between one-third and half of residents live with arthritis or heart disease, and almost half have moderate to severe cognitive impairment (Zimmerman et al., 2014). From a clinical perspective, many residents live with multiple chronic illnesses such as cardiovascular disease, diabetes, arthritis, and respiratory conditions (McNabney et al., 2014). These conditions require ongoing monitoring and careful management yet ALF settings often lack the continuous clinical oversight available in more medically intensive environments. The widespread use of multiple medications further adds to this complexity, increasing the risk of adverse drug events, drug–drug interactions, and challenges with adherence (Maher et al., 2013). Traditional single-disease clinical guidelines often fall short when applied to individuals with multiple coexisting conditions (AGS, 2012). As a result, early signs of clinical deterioration can be subtle and easily missed, especially in ALF settings where care is commonly delivered in a less IPCP centric manner by staff with varying levels of training and clinical expertise.

Clinical complexity among residents diagnosed with chronic obstructive pulmonary disease (COPD) for example, require an increase of direct care time of approximately 105 additional minutes compared to those without COPD (McNabney et al., 2014). Residents with these high acuity illnesses often require care that overburdens limited ALF staff. Mental health concerns can exacerbate co-morbid cognitive problems as well as other physical problems (Bar-tur, 2021). Because of this, ALFs must maintain staffing models and interdisciplinary care structures capable of accommodating the full diversity of resident population needs. Without adequate resources and collaborative care coordination, facilities risk being unable to retain or appropriately care for residents as their conditions progress, impeding person-centered continuity of care.

Cognitive impairment represents a critical layer of complexity. A substantial proportion of ALF residents experience mild cognitive impairment or dementia (Mitchell and Kemp, 2000), often accompanied by behavioral and psychological symptoms such as agitation, confusion, wandering, and resistance to care (Zimmerman et al., 2014). These symptoms not only increase safety risks but also make routine caregiving more challenging. Managing these issues effectively requires more than medical treatment alone; it calls for a comprehensive approach that integrates behavioral strategies, environmental adaptations, and psychological support (Kales et al., 2015).

A critical review of the literature reveals significant gaps. Assisted living facilities face notable challenges in meeting the needs of residents with dementia. ALFs can at times fail to deliver on their core promise of promoting independence and autonomy. While person-centered care is essential for maintaining independence, ALF settings frequently struggle with low staffing ratios, inadequate dementia-specific training, and funding constraints. In many cases this can lead to a culture where safety concerns override resident autonomy (Smith et al., 2021). The disconnects between resident needs and systemic capacity create an urgency for care models that maximize effectiveness of existing staff. IPCP has emerged as one such strategy, yet its application within ALFs remains under-researched and under-implemented into practice.

## Rationale for IPCP as a possible response

Addressing evolving ALF demands necessitates a fundamentally collaborative approach rather than mere incremental changes. In ALF settings, IPCP involves fostering strategic educational and practice-focused collaboration among nurses, social workers, physical therapists, prescribers, administrators, dietary staff, and other care providers to meet residents’ multifaceted needs (Reeves et al., 2018). Although IPCP is commonly recognized for its value in more medically intensive settings, it remains underutilized in ALFs and actualized in the regular and effective IPCP (Thistlethwaite, 2011).

IPCP is increasingly recognized as an integral strategy for enhancing the quality of care in assisted living settings. IPCP is utilized to align staff on daily care goals, and to promote better communication between staff and care recipients in support of a person-centered approach to care (Lin et al., 2022). This is an important aspect of long-term care that supports a collaboration focused on care recipients in healthcare settings, whereby a team from multiple disciplines can learn from each other and intentionally collaborate to provide more effective care outcomes for particular patients and more broadly as learning and effective care collaboration is translated to all facility ALF care recipients over time.

In practice, IPCP creates a collaborative learning and practice staff culture where disciplines involved can discuss clinical findings, coordinate care plans, and develop a holistic, person-centered approach. IPCP supports a collaborative caring approach in healthcare settings where health and social care disciplines provide a consistent holistic approach to care. It provides staff and healthcare providers with the tools they need to understand how behaviors or symptoms may be linked to higher severity of illnesses and conditions that could arise. Understanding the interconnectedness between disciplines can support a collaborative effort in ALFs and other healthcare settings. Providing a team approach to person-centered care is often considered a gold standard of care because effective IPE and IPCP can lead to better collaborative practice, improving healthcare systems and patient centered care outcomes (Azzam et al., 2021). Imbedding IPCP into team collaboration in ALF’s can transform the patient experience and advance iterative learning and care improvement that maximizes resident wellbeing, safety, and quality outcomes.

Equally important are the psychosocial and emotional dimensions of care. For many older adults, assisted living is not a temporary placement, but rather it ends up being a long-term home environment (Kilaberia, 2020). Maintaining meaningful social connections, emotional wellbeing, and a sense of dignity becomes central to their quality of life. Yet many residents experience loneliness, depression, or anxiety, particularly as they face declining health or loss of independence (Courtin and Knapp, 2015). Care planning, therefore, must carefully balance clinical needs with resident preferences and family expectations.

Finally, frequent transitions between care settings add another layer of complexity that IPCP can help address. Residents often move between hospitals, rehabilitation centers, and ALFs, creating multiple opportunities for communication breakdowns. These transitions are well known to be high-risk periods for medication errors, information loss, and adverse events (Elliot et al., 2020; Kandiah et al., 2023). As ALFs increasingly care for higher-acuity populations, their ability to manage these transitions safely and effectively has become a critical determinant of overall care quality that requires increased critical attention and collaborative effort.

## Practical barriers to IPCP implementation

The structural realities of ALF’s include reliance on state regulation that mandates basic supervision and personal care rather than skilled nursing (Zimmerman et al., 2022). This means ALF’s have less regulatory restrictions with federal staffing mandates. Both ALF and skilled nursing present a rationale and a barrier to IPCP integration. More than half of residents in ALFs require assistance with three or more ADLs. The majority need assistance with bathing, ambulation, and dressing (Melekin et al., 2024). This suggests that ALF’s support a higher level of care than originally intended for. This reveals limitations that IPCP can help more effectively address. ALF’s are operating under a regulatory system designed for basic care and serve a population with increasingly higher medical acuity that requires more interprofessionally coordinated care.

Another barrier in healthcare broadly and ALFs in particular is the shortage among health professionals with geriatric training. Less than 5% of professionals working in geriatric settings hold certifications in geriatrics (Flaherty and Bartels, 2019). This reality reveals a great opportunity for aging-centric IPE training, as there is a critical workforce gap in geriatric expertise across healthcare disciplines. Educating interdisciplinary team members to have a more informed and impactful understanding of the needs of the geriatric population remains an important opportunity in ALF facilities.

When implementing IPCP, there are several hurdles to overcome. Logistical challenges cause difficulty in coordinating learning activities across disciplines, especially related to other time and performance burdens (Patel et al., 2025). These scheduling difficulties are not just a simple inconvenience. They are a systemic symptom of complex differences in responsibilities, schedules, and disciplinary roles (Juntunen et al., 2024). This is a multifaceted issue that requires a specific person who is tasked with coordinating learning and navigating schedules across disciplines. Working closely with the interdisciplinary team, this dedicated person will help to create a learning schedule that makes face-to-face sessions possible (Schapmire et al., 2018). Institutional silos create another barrier. Often professional hierarchies and administrative cultures obstruct effective collaboration (Schapmire et al., 2018). These hierarchies and cultures can affect the way staff operate. They may work independently rather than as part of a comprehensive and integrated team approach. Disciplinary hierarchies can stifle communication and collaboration as valuable insights from direct care staff may often be overlooked. Oftentimes these caregivers are ignored for clinical or administrative purposes. When disciplines cannot work together because they view their role as superior, this can also lead to obstacles with team collaborative efforts.

## Measurable resident-centered outcomes that IPCP might influence

Although there are barriers to implementing IPCP, the benefits outweigh these issues. The theoretical benefits of IPCP are well-documented. Proponents argue that it fosters collaborative efforts, facilitates knowledge transfer across disciplines, and cultivates positive attitudes among team members, ultimately leading to improved communication and reduced errors (Saragih et al., 2024). The Interprofessional Curriculum for the Care of Older Adults (iCCOA) verifies that bringing together individuals that work as part of an interdisciplinary team, improve their ability to work in teams to provide a holistic, person-centered approach to care for older adults (Schapmire et al., 2018). IPCP has also been shown to be an effective approach necessary for collaborative approach to care by providing a clearer understanding of roles and responsibilities, enhanced teamwork dynamic and improved interprofessional communication (Patel et al., 2025). In ALFs institutional silos can cause historically fragmented care. IPCP can offer a way to break down those barriers. As interprofessional team members learn together, they are better prepared to work together. This can change the relationship from parallel practice to genuine collaboration.

IPCP can potentially mediate improved communication leading to improved clinical impacts. Measurable resident outcomes that IPCP might influence include improvements in interprofessional coordination, including time from symptom onset to clinical detection and intervention for common medical problems such as infections or dehydration, for example, (Nilormi et al., 2024; Shirley et al., 2021).

## Proposed operational framework

To reduce the gap in implementing IPCP with ALFs and helping facilities shift to continuously providing IPCP experience for each ALF resident, through minimal burden and practice-based collaboration, it would be beneficial to introduce to ALFs a brief practical IPCP framework (Table 1). Essentially, the IPCP framework would be an ongoing integrated process of IPCP that involves collaboration with/between the practitioners (nurses, prescribers, pharmacists, rehab specialists, and social workers) to establish and maintain structures for collaborative discussion among all practitioners, to support the earlier recognition of residents’ clinical declines, changes in function and psychosocial needs.

**TABLE 1:** Proposed donabedian-informed framework for planning, implementing, and evaluating IPE and IPCP in assisted living facilities.

Framework component	Key elements	Purpose in assisted living
Context: Resident complexity and clinical risk	Multimorbidity; cognitive impairment/dementia; polypharmacy; functional decline; fall risk; psychosocial needs; changing resident acuity	Establishes the clinical and functional complexity that creates the need for coordinated interprofessional care
Structure: Organizational readiness for IPCP	Leadership support; adequate staffing; protected learning time; educational resources; workforce mix; supportive organizational culture; access to technology/resources	Creates the organizational conditions necessary to introduce and sustain IPE/IPCP
IPCP implementation: Integration into routine practice	Brief interprofessional learning sessions; resident case discussions; team huddles; joint problem-solving; simulation or skills-based learning; reflection and feedback	Embeds IPCP into existing workflows using practical, low burden approaches rather than creating separate educational requirements
Process: Interprofessional collaborative care	Communication across roles; role clarity; shared assessment; shared decision-making; coordinated interventions; monitoring and follow-up; resident and family engagement	Translates interprofessional learning into changes in everyday team behaviors and resident care practices
Outcomes: Resident, staff, and organizational effects	Medication optimization; earlier recognition of clinical deterioration; reduced fall risk; prevention/earlier detection of dehydration and infection; fewer avoidable hospital transfers; preservation of functional capacity; improved quality of life; improved teamwork and staff/resident satisfaction	Represents the anticipated clinical, functional, workforce, and quality-of-care effects of stronger collaborative practice
Implementation evaluation	Reach; participation; feasibility; acceptability; fidelity; sustainability	Determines whether IPE/IPCP reaches intended staff, can be integrated into routine workflows, is delivered as intended, is acceptable to staff, and can be maintained over time

(Donabedian, 1988).

This will provide an ongoing practice integrated approach to improve interprofessional collaborative practice (IPC), improve communication among all professionals involved in care delivery, create clear roles for the professionals (who will be providing care) and provide the team with more time for early and timely interventions for their vulnerable residents. The first step in the integrated IPCP framework would be performing a comprehensive resident assessment and have team members communicate with each other about assessment data and information related to the resident, collaboratively make decisions based on each team member’s input, proceed to develop a comprehensive care plan together and follow through with the coordinated and implemented interventions.

The proposed IPCP framework is informed by Donabedian’s Structure–Process–Outcome model, in which organizational structures, such as staffing, leadership support, educational resources, and a culture of collaboration, provide the foundation for implementing IPCP in assisted living facilities (Donabedian, 1988). IPCP strategies, including team-based learning, resident case discussions, and interdisciplinary huddles, may strengthen care processes such as communication, role clarity, shared decision-making, and care coordination, which may subsequently contribute to improved resident, staff, and organizational outcomes. Because these outcomes represent anticipated rather than established effects of IPCP in assisted living, the framework also emphasizes evaluation of implementation, feasibility, acceptability, fidelity, and sustainability and highlights the need for future empirical research. Resident complexity and clinical risk are conceptualized as contextual drivers that influence the entire framework rather than as a sequential stage (Kobayashi et al., 2011). Implementation evaluation occurs across the IPCP initiative and is distinct from resident, staff, and organizational outcomes.

Potential benefits may include improved physiological stability, earlier recognition of changes in health status, fewer avoidable hospitalizations, preservation of functional capacity, and improved quality of life. These outcomes are particularly relevant in assisted living facilities, where maintaining residents’ health, function, independence, and safety is a central goal. However, these benefits should be considered potential rather than established outcomes of IPCP. Evidence directly linking IPCP implementation to clinical, physiological, and functional outcomes among assisted living residents remains limited. Future implementation and intervention studies are therefore needed to determine whether IPCP improves collaborative care processes and whether these improvements translate into measurable resident outcomes. The proposed framework provides a foundation for such research by identifying organizational structures, implementation strategies, collaborative care processes, and resident-centered outcomes that can be systematically evaluated.

## Discussion

Interdisciplinary education in programs related to healthcare, such as rehabilitation, nursing and public health, form a critical part of quality and effective delivery of healthcare in ALFs. Evidence has begun to emerge indicating that ALFs are experiencing higher levels of acuity among ALF resident populations due to multimorbidity, as well as experiencing a deterioration in cognitive and physical function among patients. Therefore, effective IPE processes leading to IPCP must occur within ALFs to not only enhance the quality and efficiency of healthcare delivery systems, but also to improve resident, family, staff and organizational outcomes. An example of a model for future IPCP research includes the modified Donabedian structure-process-outcomes framework (Shippee et al., 2025). This framework allows for the identification of the influencing factors of IPE and IPCP process implementation at the ALF organizational level. Examples of structural-related factors affecting the effectiveness of IPE and IPCP processes include the number of interdisciplinary team members involved in patient care as well as the types of professional disciplines represented, support of organizational leadership to include IPE, regulatory compliance requirements for IPE, availability of organizational resources (patient and staffing needs) and level of patient complexity. Application of IPE to enhance team communication, define team roles, increase collaboration in decision making, enhance care coordination and effectively manage transitions from one healthcare provider to another can greatly reduce and/or eliminate acute care hospitalizations, improve chronic disease management, improve family satisfaction, enhance staff satisfaction and improve overall quality of care within ALFs. Future research should continue to examine IPCP as a mediator for resident outcomes as well as the influence of ALF characteristics such as availability of organizational resources, type of regulatory body, type of staff employed and facility characteristics on the ability of IPCP models to positively impact outcomes. Longitudinal studies using an explanatory model that incorporates quantitative and qualitative methods to determine the impact of long-term IPE integration into team culture, team communication and resident-centered care would provide a basis to understand how continued IPE and IPCP integration improves on-going team effectiveness and the delivery of resident-centered care as it pertains to the evolving complexities of ALFs.

## Interprofessional education as a potential clinical intervention with associated links to physiological and functional outcomes in older adults

Interprofessional Education (IPE) could be seen not as merely an organizational intervention, but as a clinically relevant intervention that could provide a unique opportunity for impacting on clinical outcomes in older adults who reside in Assisted Living Facilities (ALFs). The increasing frailty and multimorbidity of residents present new opportunities for increased vulnerability to clinical decline among residents at ALFs due to complex communication networks and poorly coordinated care between health providers. IPCP combined with improved education has the potential to improve the recognition and referral of residents for medical evaluation for hydration status, infection, delirium, Medication Related Complications (MRC), or Decline in Lung Function (DLF). Improved education also has the potential to improve the timelines of intervention and subsequently improve the risk of hospitalization.

Additionally, the collaborative approach to care can optimize Medication Management and Preserve Functional Mobility and Prevent Falls, as well as develop a coordinated approach to managing chronic diseases through collaboration among Health Providers (nurses, prescribers, pharmacists, rehabilitation specialists, social workers), with direct patient care support staff. Due to the compromised health of many older adults at ALFs, many present with atypical symptoms and are highly vulnerable to health deterioration. As such, earlier, multi-discipline interventions may help residents to preserve their functioning and health as they age in place. Therefore, future studies of IPCP should explore its potential to impact the clinical placements, and the Physiological and functional outcomes associated with health services for older adults living in ALFs.

It could also be beneficial to integrate IPCP approaches in ALF settings within the context of established national and international organizational frameworks. For example, ALF’s could promote greater interprofessional integration and success by using established competencies outlined by the American Interprofessional Health Collaborative (AIHC) and the Canadian Interprofessional Health Collaborative (CIHC) as a strategy to guide IPCP practice in ALF settings (American Interprofessional Health Collaborative, 2026; Canadian Interprofessional Health Collaborative, 2024). The CIHC competency domains are particularly salient related to ALFs in that they emphasize relationship-oriented care, team communication, role clarity and negotiation, team performance, managing disagreement, and collaboration in leadership decision making (Canadian Interprofessional Health Collaborative, 2024).

In conclusion, IPE and IPCP remain both educational and organizational opportunities with clinical relevance to care interventions that can influence health outcomes among older adult residents in ALF settings. This synthesis of associated research literature identified gaps, such as the potential to make clearer opportunities to enhance ALF care through interprofessional strategies as highlighted in the proposed conceptual IPE and IPCP integration workflow. With earlier and more impactful integration of IPE and IPCP, identification and management of health issues among increasingly frail residents can measurably improve. Collaborative approaches can better optimize medication management, hydration, detection of infections, delirium, or other functional changes, and promote the prevention of falls, reductions in hospitalizations, and better chronic disease management that remain critical continued opportunities in ALF settings.

## Data Availability

The original contributions presented in the study are included in the article/supplementary material, further inquiries can be directed to the corresponding authors.

## References

[B1] American Geriatrics Society Expert Panel on the Care of Older Adults with Multimorbidity (2012). Patient-centered care for older adults with multiple chronic conditions: a stepwise approach from the American geriatrics society: American geriatrics society expert panel on the care of older adults with multimorbidity. J. Am. Geriatrics Soc. 60 (10), 1957–1968. 10.1111/j.1532-5415.2012.04187.x 22994844 PMC4459791

[B2] American Interprofessional Health Collaborative (2026). About AIHC. Available online at: https://aihc-us.org/about-aihc.

[B3] Azzam M. Puvirajah A. Girard M.-A. Grymonpre R. E. (2021). Interprofessional education relevant accreditation standards in Canada: a comparative document analysis. Hum. Resour. Health 19 (1), 66. 10.1186/s12960-021-00611-1 33985513 PMC8120702

[B4] Bar-Tur L. (2021). Fostering well-being in the elderly: translating theories on positive aging to practical approaches. Front. Med. 8, 517226. 10.3389/fmed.2021.517226 33898472 PMC8062922

[B5] Boersma P. Black L. I. Ward B. W. (2020). Prevalence of multiple chronic conditions among US adults, 2018. Preventing Chronic Disease 17, E106. 32945769 10.5888/pcd17.200130 PMC7553211

[B6] Canadian Interprofessional Health Collaborative (2024). CIHC competency framework for advancing collaboration. Available online at: https://cihc-cpis.com/wp-content/uploads/2024/06/CIHC-Competency-Framework.pdf.

[B7] Courtin E. Knapp M. (2015). Social isolation, loneliness and health in old age: a scoping review. Health and Soc. Care Community 25 (3), 799–812. 10.1111/hsc.12311 26712585

[B8] Crowley R. Atiq O. Hilden D. Beachy M. W. Brislen H. C. Curry W. A. (2022). Longterm services and supports for older adults: a position paper from the American college of physicians. Ann. Intern. Med. 175 (8), 1172–1174. 10.7326/m22-0864 35816710

[B9] Donabedian A. (1988). The quality of care: how can it be assessed? Jama 260 (12), 1743–1748. 10.1001/jama.1988.03410120089033 3045356

[B10] Eggersdorfer M. Akobundu U. Bailey R. L. Shlisky J. Beaudreault A. R. Bergeron G. (2018). Hidden hunger: solutions for America’s aging populations. Nutrients 10 (9), 1210. 10.3390/nu10091210 30200492 PMC6165209

[B11] Elliott R. A. Chan A. Godbole G. Hendrix I. Pont L. Sfetcopoulos D. (2020). Standard of practice in geriatric medicine for pharmacy services. J. Pharm. Pract. Res. 50 (1), 82–97. 10.1002/jppr.1636

[B12] Flaherty E. Bartels S. J. (2019). Addressing the community‐based geriatric healthcare workforce shortage by leveraging the potential of interprofessional teams. J. Am. Geriatrics Soc. 67 (S2), S400–S408. 10.1111/jgs.15924 31074849

[B13] Harris-Kojetin L. Sengupta M. Park-Lee E. Valverde R. Caffrey C. Rome V. (2016). Long-term care providers and services users in the United States: data from the national study of long-term care providers. Available online at: https://stacks.cdc.gov/view/cdc/157096/cdc_157096_DS1.pdf. 27023287

[B14] Juntunen J. Tuomikoski A.-M. Pramila‐Savukoski S. Kaarlela V. Keinänen A.-L. Kääriäinen M. (2024). Healthcare professionals’ experiences of required competencies in mentoring of interprofessional students in clinical practice: a systematic review of qualitative studies. J. Adv. Nurs. 81 (2), 701–729. 10.1111/jan.16347 39099212 PMC11729577

[B15] Kales H. C. Gitlin L. N. Lyketsos C. G. (2015). Assessment and management of behavioral and psychological symptoms of dementia. BMJ 350, h369. 10.1136/bmj.h369 25731881 PMC4707529

[B16] Kandiah J. Nazar H. Blacklock J. Robinson A. Wright D. (2023). Contextual factors influencing medicines-related interventions to support safe transitions for care home residents post hospital discharge: a systematic review and meta-ethnographic synthesis. Int. J. Clin. Pharm. 45, 26–37. 10.1007/s11096-022-01507-3 36394785 PMC9938806

[B17] Kilaberia T. R. (2020). Organizational commitment among residential care workers. J. Aging Stud. 55, 100894. 10.1016/j.jaging.2020.100894 33272454

[B19] Kobayashi H. Takemura Y. Kanda K. (2011). Patient perception of nursing service quality; an applied model of Donabedian’s structure-process-outcome approach theory. Scand. J. Caring Sci. 25 (3), 419–425. 10.1111/j.1471-6712.2010.00836.x 21058969

[B20] Lin S. P. Chang C.-W. Wu C.-Y. Chin C.-S. Lin C.-H. Shiu S.-I. (2022). The effectiveness of multidisciplinary team huddles in healthcare Hospital-based setting. J. Multidiscip. Healthc. 15, 2241–2247. 10.2147/jmdh.s384554 36225857 PMC9549805

[B21] Maher R. L. Hanlon J. Hajjar E. R. (2013). Clinical consequences of polypharmacy in elderly. Expert Opin. Drug Saf. 13 (1), 57–65. 10.1517/14740338.2013.827660 24073682 PMC3864987

[B22] McNabney M. K. Onyike C. Johnston D. Mayer L. Lyketsos C. Brandt J. (2014). The impact of complex chronic diseases on care utilization among assisted living residents. Geriatr. Nurs. 35 (1), 26–30. 10.1016/j.gerinurse.2013.09.003 24139207 PMC3977595

[B23] McSweeney-Feld M. H. Oetjen R. M. (2012). Dimensions of long-term Care Management: An Introduction. Chicago: Health Administration Press; AUPHA Press.

[B24] Melekin A. Sengupta M. Caffrey C. Caffrey C. (2024). Residential Care Community Resident Characteristics: United States, 2022. 10.15620/cdc/158327 PMC11616098 39302640

[B25] Mitchell J. M. Kemp B. J. (2000). Quality of life in assisted living homes: a multidimensional analysis. J. Gerontology Ser. B Psychol. Sci. Soc. Sci. 55 (2), P117–P127. 10.1093/geronb/55.2.p117 10794190

[B26] Moccia Michelle Keyes D. C. (2021). Improving care transitions: an initiative between the emergency department and senior care facilities. Spartan Med. Res. J. 6 (2). 10.51894/001c.26862 PMC8405283 34532624

[B27] Mowbray F. Ellis B. Schumacher C. Heckman G. de Wit K. Strum R. P. (2023). The association between frailty and a nurse-identified need for comprehensive geriatric assessment referral from the emergency department. Can. J. Nurs. Res. 55 (3), 404–412. 10.1177/08445621221144667 36632010 PMC10416548

[B28] Nilormi A. Heer C. Ziegler E. (2024). Developing a comprehensive curriculum program for nurse practitioners delivering primary care in the long-term care setting. Healthc. Manag. Forum 37 (1_Suppl. l), 62S–67S. 10.1177/08404704241259900 39194281 PMC11360272

[B29] Patel H. Perry S. Badu E. Mwangi F. Onifade O. Mazurskyy A. (2025). A scoping review of interprofessional education in healthcare: evaluating competency development, BMC Med. Educ. 25 (1), 409. 10.1186/s12909-025-06969-3 40114152 PMC11924666

[B30] Reeves S. Pelone F. Harrison R. Goldman J. Zwarenstein M. (2018). Interprofessional collaboration to improve professional practice and healthcare outcomes. Cochrane Database Syst. Rev. 6 (8), CD000072. 10.1002/14651858.cd000072 28639262 PMC6481564

[B31] Saragih I. D. Hsiao C.-T. Fann W.-C. Hsu C.-M. Saragih I. S. Lee B.-O. (2024). Impacts of interprofessional education on collaborative practice of healthcare professionals: a systematic review and meta-analysis. Nurse Educ. Today 138, 106176. 10.1016/j.nedt.2024.106176 38422794

[B32] Schapmire T. J. Head B. A. Nash W. A. Yankeelov P. A. Furman C. D. Wright R. B. (2018). Overcoming barriers to interprofessional education in gerontology: the interprofessional curriculum for the care of older adults. Adv. Med. Educ. Pract. 9, 109–118. 10.2147/AMEP.S149863 29497345 PMC5819582

[B33] Shippee T. P. Akosionu-DeSouza O. Beebe T. J. Parikh R. R. Brasure M. (2025). Measurement of quality in assisted living in the United States of America: a scoping review. J. Am. Med. Dir. Assoc. 26 (1), 105355. 10.1016/j.jamda.2024.105355 39551082

[B34] Shirey M. R. Selleck C. S. White‐Williams C. Talley M. Harper D. C. (2021). Interprofessional collaborative practice model to advance population health. Popul. Health Manag. 24 (1), 69–77. 10.1089/pop.2019.0194 32074013

[B35] Smith M. Brown M. Ritchie L. Papadopoulou C. Tolson D. (2021). Living with dementia in supported housing: a systematic review and thematic synthesis of qualitative research. Health and Soc. Care Community 30 (3). 10.1111/hsc.13618 34708451

[B36] Thistlethwaite J. (2011). Interprofessional education: a review of context, learning and the research agenda. Med. Educ. 46 (1), 58–70. 10.1111/j.1365-2923.2011.04143.x 22150197

[B37] Trainum K. Liu J. Hauser E. Xie B. (2024). Nursing staff’s perspectives of care robots for assisted living facilities: systematic literature review. Jmir Aging 7, e58629. 10.2196/58629 39283666 PMC11443223

[B38] Trinkoff A. M. Yoon J. M. Storr C. L. Lerner N. Yang B. K. Han K. (2020). Comparing residential long-term care regulations between nursing homes and assisted living facilities. Nurs. Outlook 68 (1), 114–122. 10.1016/j.outlook.2019.06.015 31427078

[B39] Wolbert P. Gariaqoza Y. Forrest A. R. W. Pandey S. K. (2025). Integrative Models of Care for Healthy Aging in the Elderly: A Narrative Review. 10.21203/rs.3.rs-6221967/v1

[B40] World Health Organization (2010). Framework for Action on Interprofessional Education and Collaborative Practice. Geneva: World Health Organization. Available online at: https://iris.who.int/handle/10665/70185. 21174039

[B41] World Health Organization (2025). Ageing and health. Ageining Health. Available online at: https://www.who.int/news-room/fact-sheets/detail/ageing-and-health.

[B42] Zimmerman S. Sloane P. D. Reed D. (2014). Dementia prevalence and care in assisted living. Health Aff. 33 (4), 658–666. 10.1377/hlthaff.2013.1255 24711328

[B43] Zimmerman S. Carder P. Schwartz L. Silbersack J. Temkin-Greener H. Thomas K. S. (2022). The imperative to reimagine assisted living. J. Am. Med. Dir. Assoc. 23 (2), 225–234. 10.1016/j.jamda.2021.12.004 34979136 PMC8826534

